# Cardiac tamponade due to right coronary artery perforation following pacemaker implantation: a case report

**DOI:** 10.1093/ehjcr/ytae343

**Published:** 2024-07-13

**Authors:** Zakaria Alaoui-Ismaili, Anika Klein, Jacob Eifer Moeller, Tommi Bo Lindhardt, Christian Hassager

**Affiliations:** Department of Cardiology, Copenhagen University Hospital—Rigshospitalet, Blegdamsvej 9, 2100 Copenhagen Ø, Denmark; Department of Cardiology, Copenhagen University Hospital—Rigshospitalet, Blegdamsvej 9, 2100 Copenhagen Ø, Denmark; Department of Cardiology, Copenhagen University Hospital—Rigshospitalet, Blegdamsvej 9, 2100 Copenhagen Ø, Denmark; Department of Cardiology, Odense University Hospital, Odense, Denmark; Department of Cardiology, Nordsjællands Hospital, Hillerød, Denmark; Department of Cardiology, Copenhagen University Hospital—Rigshospitalet, Blegdamsvej 9, 2100 Copenhagen Ø, Denmark

**Keywords:** Case report, Pacemaker implantation, Right coronary artery perforation, Cardiac tamponade, Active-fixation (screw-in) lead

## Abstract

**Background:**

Cardiac tamponade due to perforation of a cardiac chamber is a rare complication occurring in only 0.3% of patients undergoing permanent pacemaker (PM) implantation. Notably, perforation of the right coronary artery (RCA) following permanent PM implantation has only been reported twice in the literature. We report a rare case of RCA perforation leading to life-threatening cardiac tamponade with symptom onset 4 days after PM implantation

**Case summary:**

A 75-year-old woman underwent permanent PM implantation without any difficulties in placing pacemaker leads and with good thresholds. Four days later, the patient was readmitted in a state of shock due to cardiac tamponade. A blood gas analysis on the bloody pericardial effusion raised suspicion of ongoing arterial bleeding. A CT scan ruled out aortic dissection; instead, the source of bleeding was identified as a perforation in the RCA, which was managed surgically.

**Discussion:**

This case highlights the necessity of coronary artery perforation being among the differential diagnoses of cardiac tamponade after PM implantation, and it stresses the usefulness of performing a blood gas analysis on the bloody pericardial effusion.

**Learning points**

- Coronary artery perforation should be among the differential diagnoses of cardiac tamponade after permanent pacemaker implantation.
- A blood gas analysis on the bloody pericardial effusion is a useful diagnostic tool to determine the origin of bleeding.
- The anatomical placement of the right atrial lead is important in limiting the risk of right coronary artery perforation.

## Introduction

Many recent national and multicentre studies have yielded insights into the safety profile of cardiac implantable electronic devices (CIED), including permanent pacemakers (PMs). Overall, CIED-related complications occur in ≅10% of patients within six months of implantation, mostly related to the transvenous leads and subcutaneous pockets.^1–4^ Among these complications, the most prevalent major adverse events include lead-related re-intervention (1.0–5.9%), pneumothorax requiring drainage (0.5–2.2%), infection (0.7–1.7%), and cardiac perforation (0.3–0.7%).^1–3^ The most frequent site of cardiac perforation is the right ventricular (RV) apex, while perforation of the right atrial appendage (RAA) is distinctly rare.^5^ A life-threatening consequence of cardiac perforation is cardiac tamponade. Cardiac tamponade occurred in 0.3% of the 922 549 patients who underwent PM implantation, according to a comprehensive American study.^6^

This case report describes a rare incident of cardiac tamponade following PM implantation due to right coronary artery (RCA) perforation by an active-fixation (screw-in) lead in the RAA occurring 4 days after implantation.

## Summary figure

**Figure:**
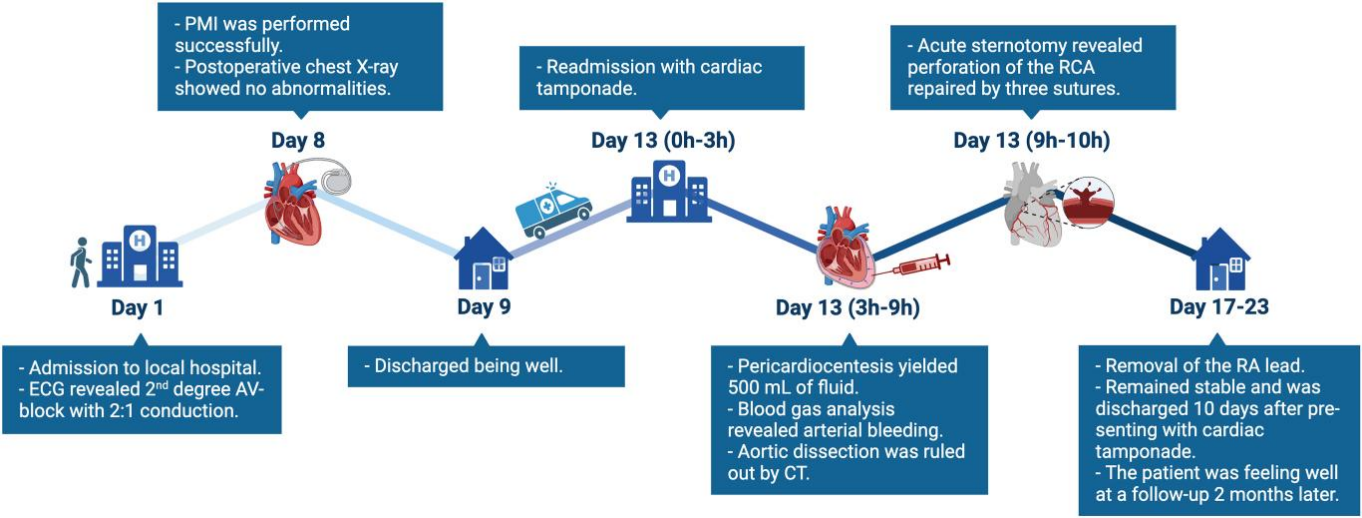


## Case presentation

**Figure 1:**
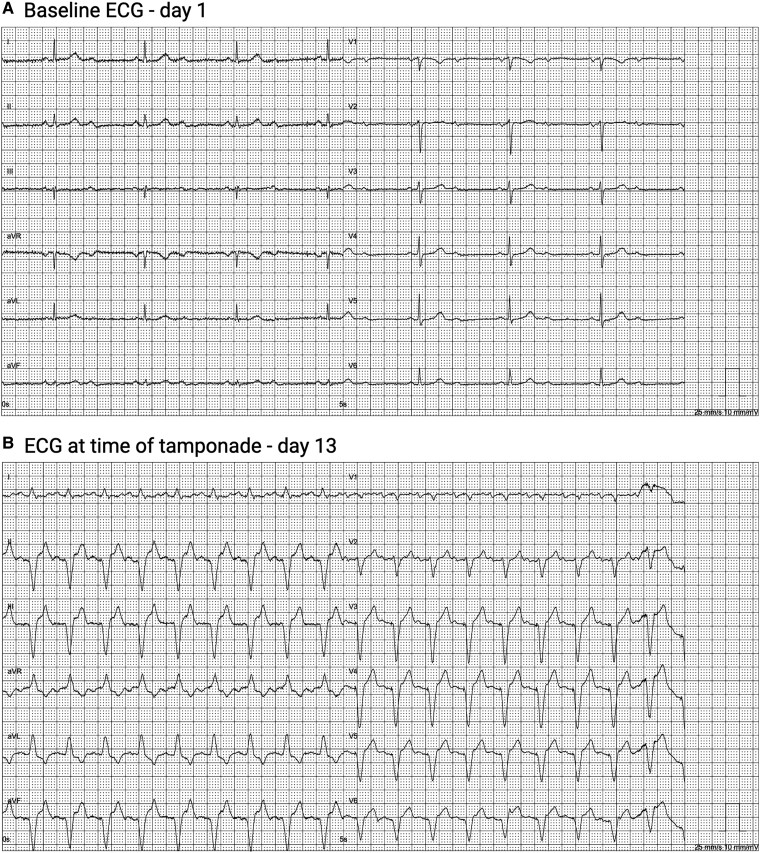
(*A*) Baseline ECG at admission revealed 2nd degree AV block with 2:1 conduction. (*B*) ECG during cardiac tamponade revealed atrial-sensed, ventricular-paced rhythm.

A 75-year-old woman with a medical history of transient cerebral ischaemia, deep vein thrombosis, and hypertension was referred to the emergency department due to bilaterally swollen legs. The list of medications included calcium 400 mg + D-vitamin 19 µG, alendronic acid 70 mg, rivaroxaban 10 mg, simvastatin 40 mg, furosemide 40 mg, amlodipine 5 mg, losartan 100 mg, and eletriptan 40 mg. Apart from a few episodes of dizziness, the patient expressed no further complaints. Echocardiography revealed normal ventricular function, and the oedema was interpreted as a side effect of antihypertensive treatment with amlodipine. An electrocardiogram (ECG) revealed 2nd degree atrioventricular block with 2:1 conduction and a heart rate of 45 beats/min (*Figure 1A*).

**Figure 2:**
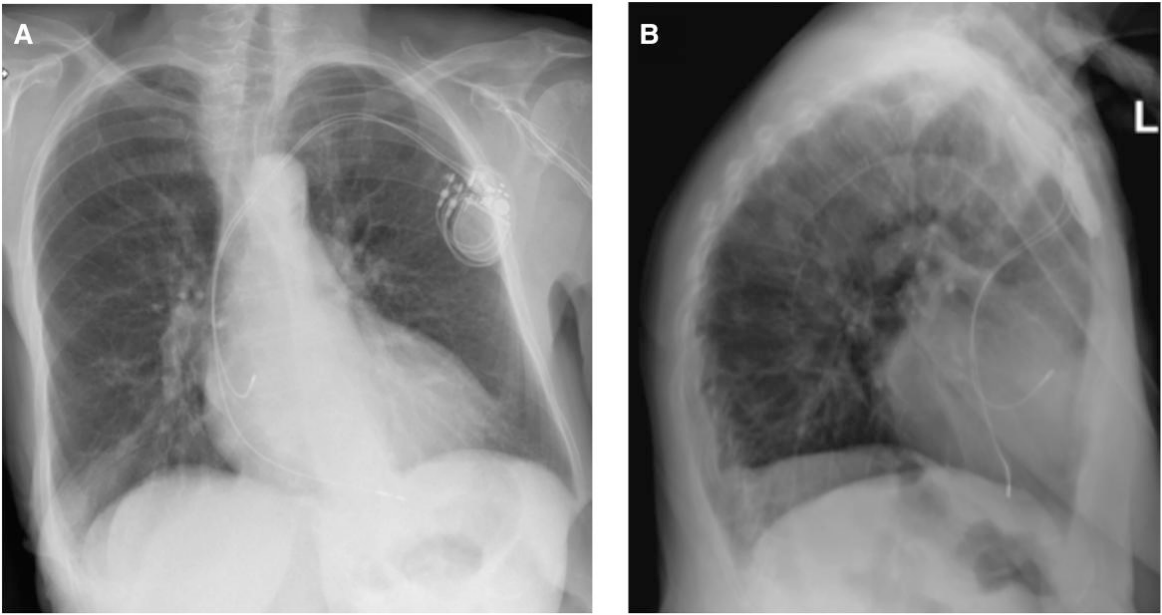
A chest X-ray following PM implantation revealed no evident abnormalities on either posteroanterior view (*A*) or lateral view (*B*).

A dual chamber (DDD-)PM with bipolar active-fixation leads placed at the RV apex and RAA, respectively, was implanted successfully through the left subclavian vein on the first attempt. A perioperative test showed normal function of the PM with appropriate pacing, sensing, and impedance. The RA pacing threshold was 0.7 V at 0.4 ms, the sensing threshold was 3.0 mV, and the impedance was 539 Ω. The RV pacing threshold was 0.7 V at 0.5 ms, the sensing threshold was 13.4 mV, and the impedance was 575 Ω. A chest X-ray (*Figure 2*) performed post-operatively showed no abnormalities, and the patient was discharged the next day feeling well.

Four days later, the patient was readmitted to the local emergency department due to weakness and shortness of breath. The patient was cold and pale with clammy skin, a low blood pressure of 60/40 mmHg, and a heart rate of 113 beats/min, consistent with shock. An ECG showed an atrial-sensed, ventricular-paced rhythm (*Figure 1B*), while echocardiography revealed pericardial effusion (1–1.5 cm) with evidence of cardiac tamponade. Conventional pacemaker interrogation revealed RV-pace of 98%, reductions in both RA and RV sensing threshold (2.3 and 9.0 mV, respectively), as well as a decrease in RA impedance to 360 Ω.

The patient was transferred to a tertiary heart centre where urgent echocardiographic-guided pericardiocentesis was performed by an experienced senior operator through an apical puncture approach, immediately yielding 500 mL of bloody fluid and quickly stabilizing the patient’s haemodynamic status. A pericardial drainage catheter was left in place, and 2 h later, there was a total leakage of 1 L of blood. Simultaneously, the patient received several blood transfusions and Xarelto® (rivaroxaban) treatment was paused and reversed with Octaplex® (prothrombin complex concentrate).

**Figure 3:**
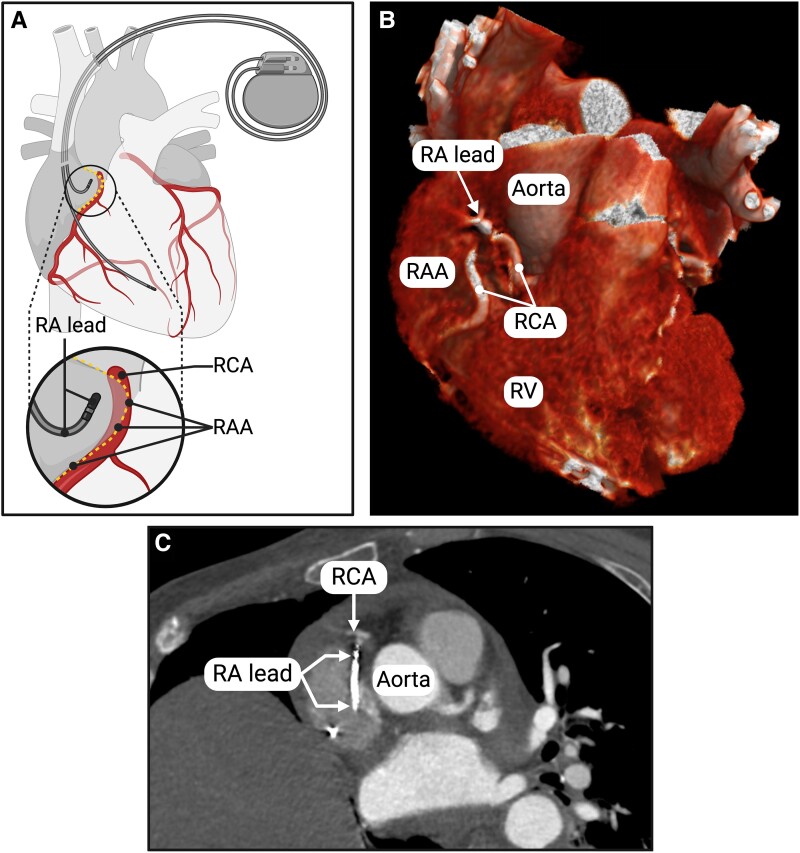
(*A*) A graphical illustration showing the possible proximity of the RCA and RA lead. (*B*) A three-dimensional volume-rendered CT image of the patient’s heart reveals that the proximal part of the RCA bends towards the RAA, coming close to the RA lead. (*C*) A modified axial CT image of the chest. RA lead, right atrial lead; RCA, right coronary artery; RAA, right atrial appendage; RV, right ventricle.

The drained pericardial fluid was bright red, and, as shown in *Table 1*, a blood gas analysis confirmed the suspicion of it being arterial blood. Computed tomography (CT) of the chest ruled out aortic dissection. Still, it revealed that the tip of the right atrial (RA) lead was in close proximity to the RCA, although perforation could not be confirmed (*Figure 3B* and *3C*).

**Table 1:** Blood gas analysis of the pericardial fluid

Value	Pericardial fluid	Blood from the radial artery (for reference)
PO_2_	9.7 kPa	11.0 kPa
PCO_2_	5.6 kPa	5.2 kPa
sO_2_	0.90	0.94
HCO_3_^−^	15.5 mmol/L	16.7 mmol/L
BE	−10.6	−9.3
Lactate	4.1 mmol/L	2.9 mmol/L
Hgb	7.6 mmol/L	8.2 mmol/L

The PO_2_, haemoglobin, and sO_2_ values confirmed the suspicion of ongoing arterial bleeding.

PO_2_, partial pressure of O_2_; PCO_2_, partial pressure of CO_2_; sO_2_, saturation of O_2_; BE, base excess.

Due to ongoing bleeding, an acute sternotomy was performed, and the source of bleeding was identified as a punctuate perforation on the proximal part of the RCA just outside the RAA. The perforation was repaired by three repeating sutures, and haemostasis was established. A perforation of the RAA could not be identified, and further thorough inspection of the pericardium and heart revealed no other bleeding.

Subsequent ECG revealed sinus rhythm with normal RV capture to the ventricles. However, a test of the PM the next day indicated that the RA lead did not capture the atrium in an unipolar setting, suggesting that there was a perforation of the RA lead tip. As the patient rarely experienced symptoms from the bradyarrhythmia, the RA lead was therefore removed, downgrading to a single-chamber (VVI-)PM.

The patient remained haemodynamically stable post-operatively and was discharged 10 days after presenting with cardiac tamponade. A follow-up two months later revealed that the patient was still being well.

## Discussion

We present a case of RCA perforation as a complication of PM implantation, resulting in life-threatening cardiac tamponade. Perforation by the RA lead was considered the most likely reason for RCA perforation, even though the lead was not visible during the acute sternotomy. This is a rare case that stands out from previously reported cases^7,8^ by the time of symptom onset (4 days following PM implantation) and the use of a blood gas analysis as a diagnostic tool.

Early recognition and treatment of cardiac tamponade is paramount to survival.^9^ Haemopericardium following PM implantation is usually of venous origin, caused by perforation in the RV apex. In this case, a quick and readily accessible blood gas analysis of the pericardial fluid revealed ongoing arterial bleeding, thus stressing the need for further immediate evaluation and treatment. This emphasizes the importance of performing a blood gas analysis every time bloody aspiration is yielded from pericardiocentesis.^10^

The sequence of the diagnostic assessment could be improved. An initial ECG recording without pacing, to detect potential ST/T abnormalities, would benefit the diagnosis of cardiac tamponade. Additionally, unipolar pacemaker interrogation should be conducted first to confirm the displacement of the RA lead before proceeding with surgical sternotomy. Surgical intervention was performed to determine and treat the bleeding aetiology. Alternatively, if coronary perforation is suspected, a coronary angiography followed by percutaneous coronary intervention (PCI) with a covered stent could be attempted, in line with the existing evidence of managing coronary artery perforation following PCI.^9,11^

The RCA runs in the atrioventricular groove covered partially by the RAA (*Figure 3A*). Accurate placement of the RA lead tip is crucial to minimize the risk of perforation. Following ESC guidelines,^3^ placing the RA lead tip in the anterior wall of the RAA is advisable. The lateral wall of RAA is particularly thin, posing an increased risk of perforation, while the medial wall of RAA is in close relation to both RCA and the root of the ascending aorta.^3,12^

In this case, an active-fixation lead was used for the PM implantation. Previous studies reveal divergent results regarding incidences of perforation with active-fixation leads compared to passive-fixation (tined) leads.^3,5,13,14^ However, in case of cardiac perforation, it is typically easily managed without cardiac surgery and with complete recovery without lasting sequelae.^5^

In conclusion, we report a rare case of permanent PM implantation leading to cardiac tamponade due to RCA perforation by an RA active-fixation lead, which distinctly differs from the more commonly recognized but still infrequent complication of cardiac tamponade due to RV perforation. This case also highlights the necessity of coronary artery perforation being among the differential diagnoses of cardiac tamponade after PM implantation, and it stresses the usefulness of performing a blood gas analysis on the bloody pericardial effusion, emphasizing the need for further immediate evaluation and treatment.

## Data Availability

The data underlying this article are available in the article.

## Lead author biography

Zakaria Alaoui-Ismaili is a pregraduate research year assistant at the Department of Cardiology, Copenhagen University Hospital—Rigshospitalet. His passion and research areas include acute cardiology, specifically cardiogenic shock and cardiac arrest. He is currently updating a retrospective register known as ‘RETROSHOCK’. This comprehensive registry consists of all patients who have experienced acute myocardial infarction complicated by cardiogenic shock, spanning the years from 2018 to 2022.
